# Right stellate ganglion block improves learning and memory dysfunction and hippocampal injury in rats with sleep deprivation

**DOI:** 10.1186/s12871-021-01486-4

**Published:** 2021-11-08

**Authors:** Dongsheng Dai, Biqiong Zheng, Zenggui Yu, Shizhu Lin, Yijie Tang, Mengnan Chen, Peng Ke, Chengjie Zheng, Yanqing Chen, Xiaodan Wu

**Affiliations:** 1 Department of Anesthesiology, Shengli Clinical Medical College, Fujian Medical University, Fuzhou, 350001 Fujian China; 2 Department of Anesthesiology, Anesthesiology Research Institute, the First Affiliated Hospital, Fujian Medical University, Fuzhou, 350001 Fujian China; 3 Fujian Provincial Clinical Medical College, Fujian Medical University, Fuzhou, 350001 Fujian China

**Keywords:** Sleep deprivation, Stellate ganglion block, Spatial learning and memory, Hippocampus, Mechanism

## Abstract

**Background:**

Sleep deprivation (SD) often leads to complex detrimental consequences, though the mechanisms underlying these dysfunctional effects remain largely unknown. We investigated whether the right stellate ganglion block in rats can improve the spatial learning and memory dysfunction induced by sleep deprivation by alleviating the damage of hippocampus in rats.

**Methods:**

Sixty four male Sprague Dawley rats were randomly divided into four groups: Control, SD (sleep deprivation), SGB (stellate ganglion block) and SGB + SD (stellate ganglion block+ sleep deprivation) (*n* = 16). The SGB and SD + SGB groups were subjected to right stellate ganglion block through posterior approach method once per day. SD and SD + SGB groups were treated with modified multi-platform water environment method for 96 h sleep deprivation in rats and their body weights were analyzed. Histopathological changes of hippocampal neurons in rats and the expression of Caspase-3 in hippocampus of rats was detected by western blotting. ELISA was used to detect the content of IL-6, IL-1 in hippocampus and serum melatonin levels.

**Results:**

Compared with the group SD, the spatial learning and memory function of the group SD + SGB was improved, the weight loss was alleviated, the pathological damage of the hippocampus was reduced and the expression of IL-6, IL-1β and Caspase-3 in the hippocampus was decreased. The content of rat serum melatonin was also increased.

**Conclusions:**

The right stellate ganglion block can improve the spatial learning and memory dysfunction of rats with sleep deprivation, and the underlying mechanism may be related to alleviating the apoptosis and inflammation of hippocampus of rats with sleep deprivation.

**Supplementary Information:**

The online version contains supplementary material available at 10.1186/s12871-021-01486-4.

## Introduction

Sleep with its various physiological and temporal stages is necessary for maintaining proper health and survival in animals and humans. Even after many decades of extensive research into the functional modalities of different stages of sleep-wake cycle, the mechanism underlying the detrimental consequences of sleep deprivation (SD) on rodents and humans has not been uncovered. However, considerable evidences were established that sleep deprivation can impair emotion, cognitive function and psychomotor performance [1–6]. The distribution of consequences can range from serious events (efficiency of high-tech jobs, special occupation security and implement of military activities, etc.) to daily performance barriers [1–3]. The influence degree of the cognition process may vary widely, such as the distribution of personal attention, reasoning, creativity, working memory, and cognitive control [4–8]. Furthermore, previous epidemiological studies have shown that people who are prone to sleep deprivation are associated with increased incidence of some diseases including obesity, diabetes, cardiovascular disease, cancer, Alzheimer’s disease, and eventual mortality [9]. Contrarily, some studies have suggested that the effect of sleep deprivation may largely depend on the time window and varying duration of SD. Few researchers have investigated that 6–12 h of short-term sleep deprivation prior to cerebral ischemia produces neuroprotective effects by attenuating inflammatory responses and glial reactions in the rat hippocampus [10–12]. Moreover, it was also reported that 12 h of short-term SD can promote neurogenesis in the hippocampus of normal rats [13, 14].

Extensive literature confirms that sleep deprivation impairs cognitive function [15]. Research by Van et al. have suggested that selective slow wave sleep deprivation can impair hippocampal coding activity [16]. Sleep deprivation can impair cognitive function by reducing prefrontal cortex task-related functional activities [17]. Hippocampal mitochondrial dysfunction such as impaired complex IV activity and increased oxidative stress is one of the important mechanisms of sleep deprivation leading to cognitive impairment [18]. In addition, neurodegeneration, microglia activation, and neuronal apoptosis will occur in the hippocampus of mice after sleep deprivation [19]. It has been confirmed in clinical and animal experiments that sleep deprivation leads to increased white blood cell counts and elevated levels of inflammatory factors such as C-reactive protein (CRP), IL1, IL6, and TNF [20].

Melatonin (N-acetyl-5-methoxy tryptamine) is a physiological hormone exclusively produced in the pineal gland of animals. During the last decades, melatonin has been widely identified and qualified in various foods from fungi to animals and plants. Several health benefits of melatonin have been documented, such as enhancing the immune system [21], showing anti-aging [22] and anti-inflammatory effects [23] and performing anticancer activities [2]. Melatonin is commonly used as a therapeutic agent for sleep disorders in individuals with a history of insomnia, and for initiating sleep and/or improving sleep efficacy [21, 24, 25]. Several meta-analyses were performed to determine the magnitude of effect in studies of melatonin in improving sleep and the results showed the most convincing evidence for exogenous melatonin use in reducing sleep onset latency in primary insomnia, delayed sleep phase syndrome, and regulating the sleep-wake patterns in blind patients compared with placebo [26].

Many studies have shown that sleep plays an important role in learning and memory function [27, 28]. Moreover, it was reported that sleep deprivation was associated with cognitive function decline and is mediated through melatonin. Studies have found that melatonin can inhibit the hypothalamic pituitary gonadal axis, decrease the level of the gonadotropin releasing hormone, and can reduce the content of androgen, estrogen and progesterone by directly acting on gonads [29].

It was previously demonstrated that various effects of stellate ganglion block (SGB) may exhibit similar effects mediated through the therapeutic intervention with melatonin [8, 25]. Stellate ganglion block can prevent the breakage of cervical sympathetic preganglionic fibers, reduce central sympathetic nerve tension, playing an important role in regulating the balance in the cardiovascular system, autonomic nervous system, endocrine system, and immune system [21–24, 30]. Based on these findings, we study the effect of SGB on learning and memory dysfunction caused by sleep deprivation. This study intends to use the behavioral platform to observe the learning and memory function of rats and histological changes of hippocampus were detected to clarify the role and action mechanism of stellate ganglion block.

In order to further evaluate the consequences of stellate ganglion block on sleep disturbance in cognitive function decline, a new sleep disturbance model in rats was established and have assessed the effects of sleep disturbance on learning and memory function in rats. Moreover, we intended to establish a novel therapy to reduce the learning and memory dysfunction caused by sleep deprivation.

## Methods

### Statement about ARRIVE guidelines

I confirming that the study is in accordance with the ARRIVE Guidelines in method section.

### Animals

Male Sprague Dawley rats, weight 220-250 g, were obtained from Laboratorial Animal Center of FuJian Medical University (FuJian, China). All animals were housed in the animal service of the laboratorial center of FuJian Medical University in Fuzhou. Before starting the behavior experiments, animals had 7 days to acclimate experimental environments, and each group of rats was placed on a sleep deprivation box platform for 2 hours a day. Room temperature was kept over 23-25 °C, under a 12 h day/night cycle [31].

Five animals were kept in a cage, and food and water were given ad libitum. Animals used in behavior experiments were grouped randomly. All experiment procedures were performed in accordance with FuJian Medical University Guideline for Care and Use of Laboratory Animals and with the approval of college ethics committee. Rats were randomly divided into group C (Control), group SD (sleep deprivation) and group SD + SGB (stellate ganglion block + sleep deprivation) (*n* = 16).

### Right stellate ganglion block

Group SGB and group SGB + SD rats were subjected to right stellate ganglion block 6 days before sleep deprivation to the end of the experiment, once a day. SGB was performed through posterior approach after sevoflurane inhalation anesthesia [32], after inserting the needle from the lateral transverse process of the seventh cervical vertebra. A little back after transverse process, 0.2% bupivacaine 0.2 ml was injected, and after anesthesia recovery, the success of the SGB was interpreted as the rats’ blocking side showing typical Horner syndrome, such as blepharoptosis, palpebral fissure narrow, and miosis and so on. The procedure was intervened once a day, with the block time at about 15:00-17:00. Rats in group SD was inserted with the same volume of normal saline following the similar procedure, and group C were not treated.

### Modified multiple platform water environment method

Sleep deprivation model was established by the modified multiple platform method (MMPM). Two homemade sleep deprivation rat boxes (110 cm*60 *40 cm) were set up with the following dimensions and conditions: Six platforms with 6.5 cm in diameter, 8 cm tall, platforms interval 15 cm, filled with water around the platform, maintained the water temperature at 22 degrees, distance from water surface to the platform was about 1 cm, rats can ingest, drink and move on the platform. When the rats entered REM sleep, the body’s muscle tension was reduced, which caused the body imbalance and rats woke up and ensured that the rat can’t enter REM sleep period. Group SGB and group C were placed in a large platform with water surrounded beside the sleep deprivation box in the same environment. Three large platform water environment mouse boxes (110 cm × 60 cm × 40 cm) were made. Two large platforms with 45 cm diameter and the same sleep stripping box were filled with water around the platform. The water temperature was kept at 22 °C and the water surface was about 1.0 cm away from the platform. The rats could move freely on the large platform, with enough drinking water and sleep.

### Morris water maze

Morris water maze (SLY-WMS water maze analysis system was purchased from Shanghai Xin Ruan Information Technology Co., Ltd.) was used to detect spatial learning and memory function of rats [31]. Every group began water maze training in the first day of sleep deprivation. Four times a day, and the time period followed between 9:00 am-10:00 am, and 15:00 pm-16:00 pm. Twice at every time period, interval time of each rat is 30S, and recorded the escape latency (the time from enter the water to find the security platform), limit test time to 60S. A camera was set up over the pool, connected to the computer and monitor, the water maze detection software system could automatically track and record the movement path and time after rats entered the pool. Experiment testing projects are placed such as the navigation test and space exploration test. Each rat was placed facing the pool wall respectively into the pool from four different entry points during the detection, and recorded the time in seconds from rats entering the water to find and stand on the hidden underwater platform, and regarded as the incubation period. Allowed the rats stand on the platform for 10S after finding the platform, its movement path was recorded at the same time and observed each group rats to find platform movement rules. After removal from the pool, rats were manually dried with a terrycloth towel and placed in a warming cage (consisting of a heating pad set to low underneath a typical shoebox cage) for at least 5 min before returning to the home cage. Rats were visually inspected to ensure thorough dryness. The following day after the last time acquisition phase, removed the platform, and began 60s probe training. Animals were placed to enter water from the opposite sides of original platform quadrant. Recorded the time of stay in the target quadrant (original platform quadrant) and the time of entering the quadrant, which was regarded as test indexes of spatial memory. Space exploration test was tested immediately after sleep deprivation to observe the spatial learning and memory effects of rats caused by sleep deprivation.

### Histological examination

At the end of Morris water maze space exploration experiment, 8 rats were randomly selected according to the computer random digital method (SPSS 20.0 software). About 3 ml of inferior vena cava blood samples were taken and placed in EP tube to detect serum melatonin content. The bilateral hippocampal tissues were rapidly isolated from the rats after blood collection, and the caspase-3 were detected by Western Blotting and ELISA respectively. The remaining eight rats in each group were given 3% pentobarbital sodium intraperitoneal injection (30 mg/kg). The heart was exposed to the chest, and the Physiological saline solution 500 ml was rapidly perfused through the left ventricle, and then continued to be perfused with 250 ml paraformaldehyde to fix the tissue and stain by HE staining.

### Detection of serum MT and hippocampal IL-6, IL-1β by ELISA

Before determination, the serum and hippocampal tissue supernatant were reconstituted in 4 °C ice water, centrifuged again by 3500 rpm for 5 min. The expression levels of serum melatonin and hippocampal IL-6 and IL-1 were measured by ELISA (ELISA Kit for Interleukin 6, ELISA Kit for Interleukin IL-1β and ELISA Kit for Melatonin (MT) were purchased from Wuhan You Er sheng Trading Company). The procedure was strictly in accordance with the kit instructions, and the absorbance (OD value) of each well was measured in sequence at 450 nm wavelength. Taking the concentration of the standard material as the longitudinal coordinate and the OD value as the transverse coordinate, the multinomial quadratic regression equation of the standard curve was calculated. The OD value of the sample was replaced by the equation, and the sample concentration was calculated, multiplied by the dilution multiple, that is, the actual concentration of the sample.

### Detection caspase-3 of hippocampus by Western blotting

Extraction of hippocampus tissue protein was performed with BCA method. Mixed 100 μg samples into 1/4 the protein volume of loading buffer, boiled for 7 min at 100 °C, incubated protein Marker in 65 °C water bath with 12% SDS polyacrylamide gel electrophoresis. The isolated protein was transferred to a PVDF membrane activated by methanol using a semi dry transfer method, sealed with 5% skimmed milk powder at 4 °C overnight, and treated with rabbit anti-phosphorylated caspase-3 polyclonal antibody (Anti-Caspase 3, Active antibody produced in rabbit was purchased from Sigma C. Ltd., USA) diluted with blocking solution (1:100 dilution) and kept shaking for 2 h at room temperature. Then, using membrane washing liquid washed for 3 times, 15 min for the first time, after two times of 10 min, with two biotin labeled antibodies (Sigma,USA) (1:14000 dilution) and were incubated for 1 h. Color developed in the samples by DAB method with beta -actin (Cell Signaling, USA) as the internal reference. The experiment was repeated 6 times. The specific protein band detected was 32ku. The software of Quantity one was used to analyze the gray value of protein bands at different time points.

### Brain tissue harvest and HE staining of hippocampus

The brain tissue taken out after perfusion was dehydrated with multi-concentration gradient sucrose at 4 °C and fixed overnight in 4% paraformaldehyde. After dehydration, wax dipping and paraffin embedding, the coronal sections of hippocampal related areas were made with 5um layer thickness. Section was subjected to dewaxing hydration, hematoxylin staining for 5 min, conventional alcohol gradient dehydration, clearing in xylene, neutral gum seal, followed by microscopic observation under 200× magnification.

### Statistical analysis

The normality of distribution was assessed with the Kolmogorov–Smirnov test. Parametric data were reported as mean (standard deviation (SD)) and non-parametric data were reported as median and interquartile range (IQR). SPSS 20.0 software was used for experimental results analysis. Repeated measures analysis of variance was used to calculate Escape latency, One-way ANOVA was used to analyze the number of crossing platforms, the percentage of target quadrant time, body weight, relative expression of caspase-3, MT, IL-1β and IL-6. Covariance analysis was used to exclude no significant effect of body weight and behavior. Those who satisfy the homogeneity of variance was subjected to the LSD test for post hoc comparison, and those who do not satisfy the homogeneity of variance are used for post hoc comparison using Dunnett’s T3. Statistical significance was defined as *P* < 0.05.

## Results

### Effect of right stellate ganglion block after sleep deprivation on spatial learning and memory ability in rats

**Fig. 1:**
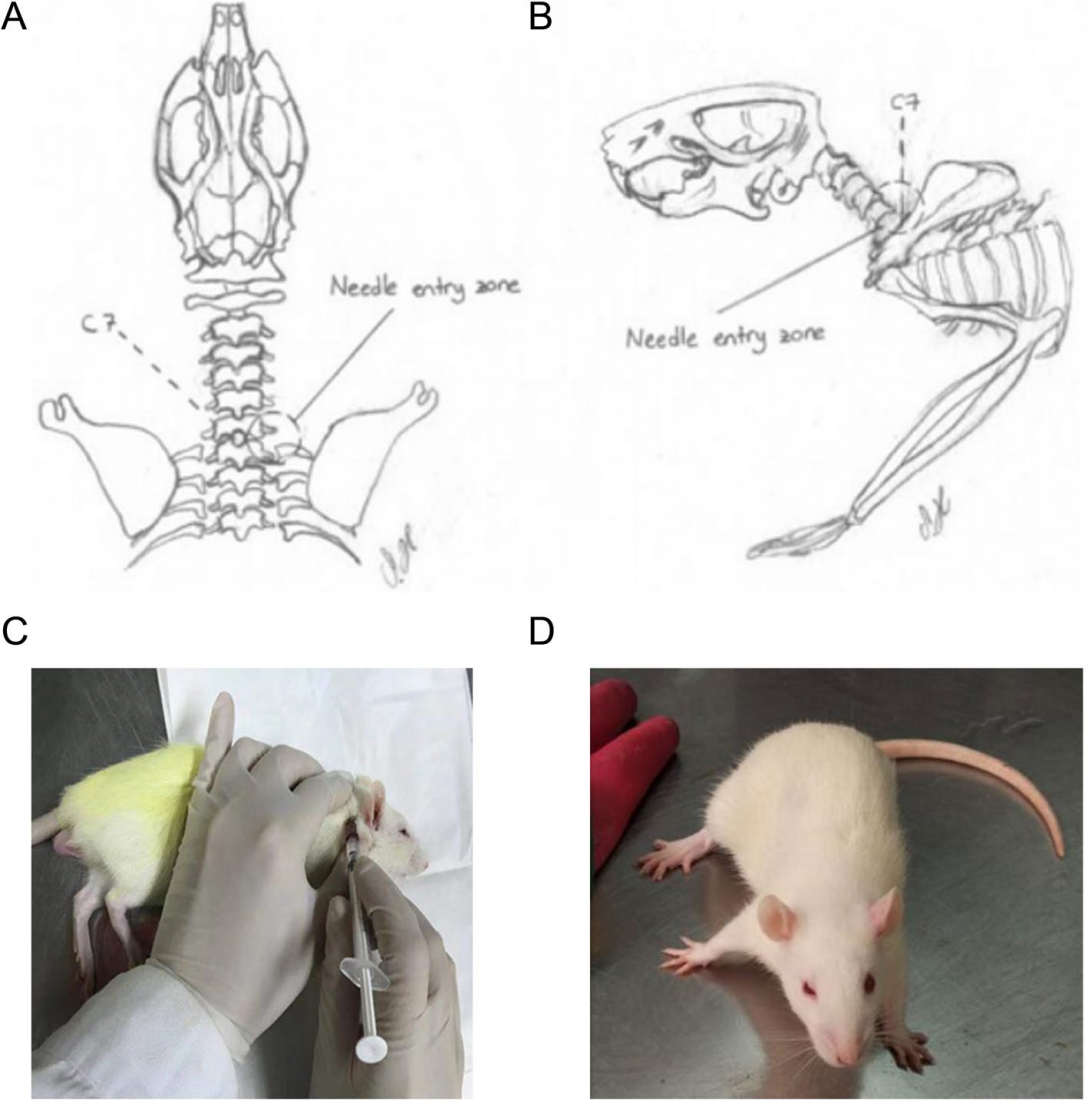
Anatomy position of rat stellate ganglia (**A** and **B**), schematic diagram of operation method of right stellate ganglion block in rat (**C**), and schematic diagram of successful rat right stellate ganglion block (**D**)

The anatomical position of rat stellate ganglion, operation method of right stellate ganglion block and the successful procedure of right stellate ganglion block are shown in Fig. 1.

**Fig. 2:**
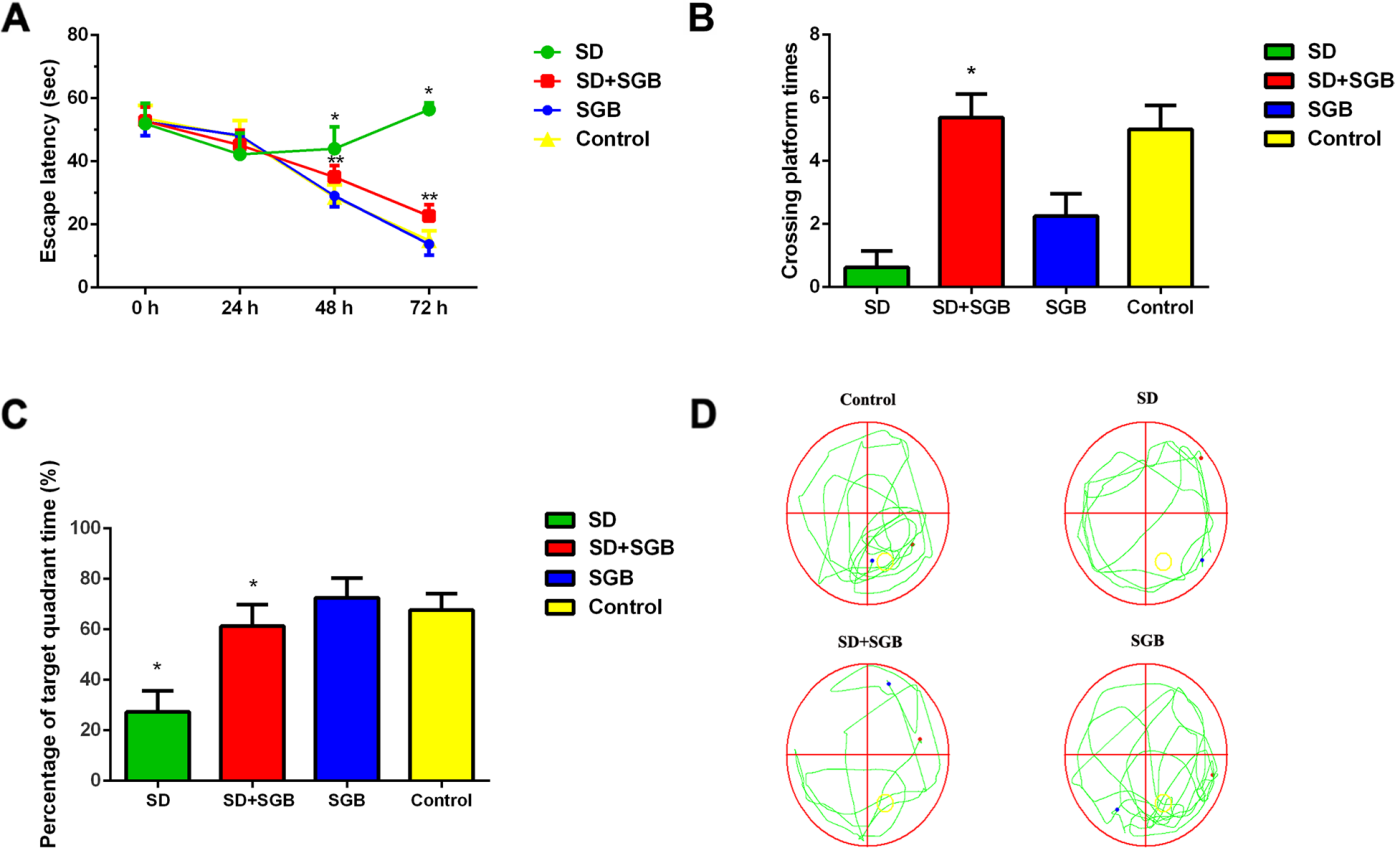
**A** Detection of escape latency in Morris water maze directional navigation test of rats in each group (*n* = 16, x ± s). Detection results of the number of crossing the platform (**B**) and the percentage of target quadrant residence time in each group of rats in the water maze space exploration experiment (**C**) (n = 16,^−^x ± s). Experimental trajectory map of water maze space exploration of rats in each group (**D**). Note: *P < 0.05, as compared with the Control group

The escape latency of the SD group was significantly prolonged compared to the Control group (*P* < 0.05). Furthermore, compared with SD group, the escape latency of rats in SD + SGB group was gradually shortened (*P* < 0.05). The change of escape latency in SGB group and Control group tends to be consistent with time (*P >* 0.05) (Fig. 2A).

The number of rats crossing the platform and the percentage of quadrant time in the original platform in SD group were significantly lower than those in control group (*P* < 0.05). Additionally, the frequency of crossing the platform and the percentage of quadrant time in the original platform in SD + SGB group were significantly higher than those in SD group (*P* < 0.05). Compared with control group, there was no significant difference in the number of rats crossing the platform and the percentage of quadrant time in the original platform in SGB group (Fig. 2B and C). Experimental trajectory map of water maze space exploration of rats in each group are shown in Fig. 2D. The platform was removed, then the resident time and frequency with which the rats crossed the target quadrant was recorded. Relative to the SD group, the rats in SD + SGB group showed significantly higher crossing times and resident time in the test (*p* < 0.05).

### Right stellate ganglion block after sleep deprivation alleviated weight loss, pro-inflammatory cytokines and serum melatonin levels in rat hippocampal tissue

**Fig. 3:**
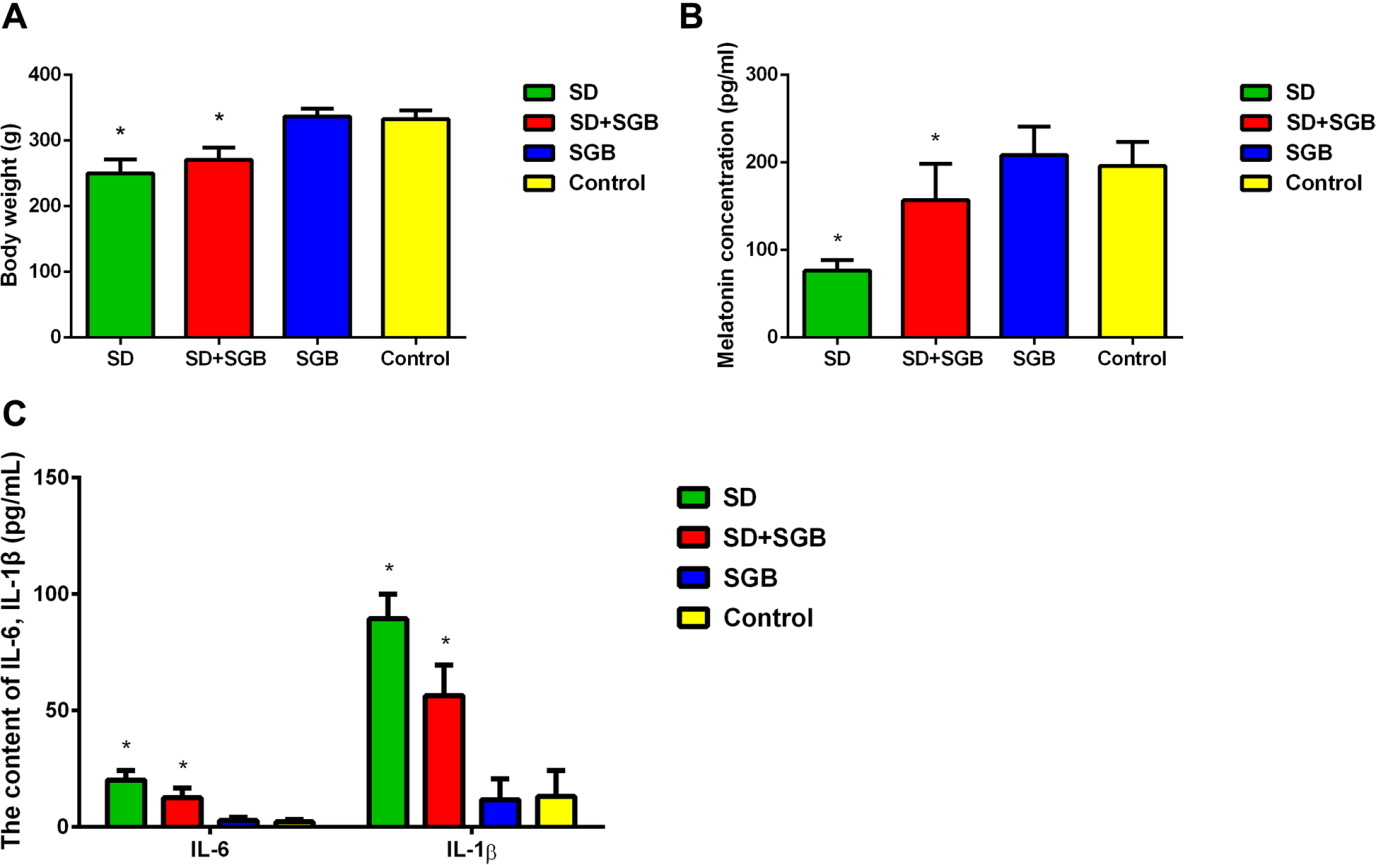
Results of body weight (**A**), serum MT content (**B**), and IL-6, IL-1 β (**C**) in hippocampal tissue of rats in each group (*n* = 8,^−^x ± s). Note: *P < 0.05, as compared with the Control group

Sleep deprivation reduced the body weight of rats compared with the control group rats (Fig. 3A). On right stellate ganglion block, the rats (SD+ SGB group) showed significantly increased body weight compared with the SD group (*P* < 0.05). Further analysis of covariance showed no significant effect on body weight compared with escape latency and number of crossing platforms (*F* = 0.037, *P* = 0.849; *F* = 0.113, *P* = 0.739).

Compared with the control group, there was no significant difference (*P* > 0.05) in IL-6, IL-1 β content and serum MT content in hippocampus of rats in SGB group (Fig. 3B and C). But the level of IL-6 and IL-1 β in hippocampal tissue of SD group were significantly higher than those of control group (Fig. 3C), while the content of serum MT was significantly lower (*P* < 0.05) than that of control group (Fig. 3B). The levels of IL-6 and IL-1 β in hippocampus of SD + SGB group were significantly lower (*P* < 0.05) than those of SD group, but the content of serum MT of SD + SGB group was significantly higher than that of SD group (Fig. 3B and C).

### Right stellate ganglion block after sleep deprivation alleviated caspase-3 mediated apoptosis of hippocampal neurons in rats

**Fig. 4:**
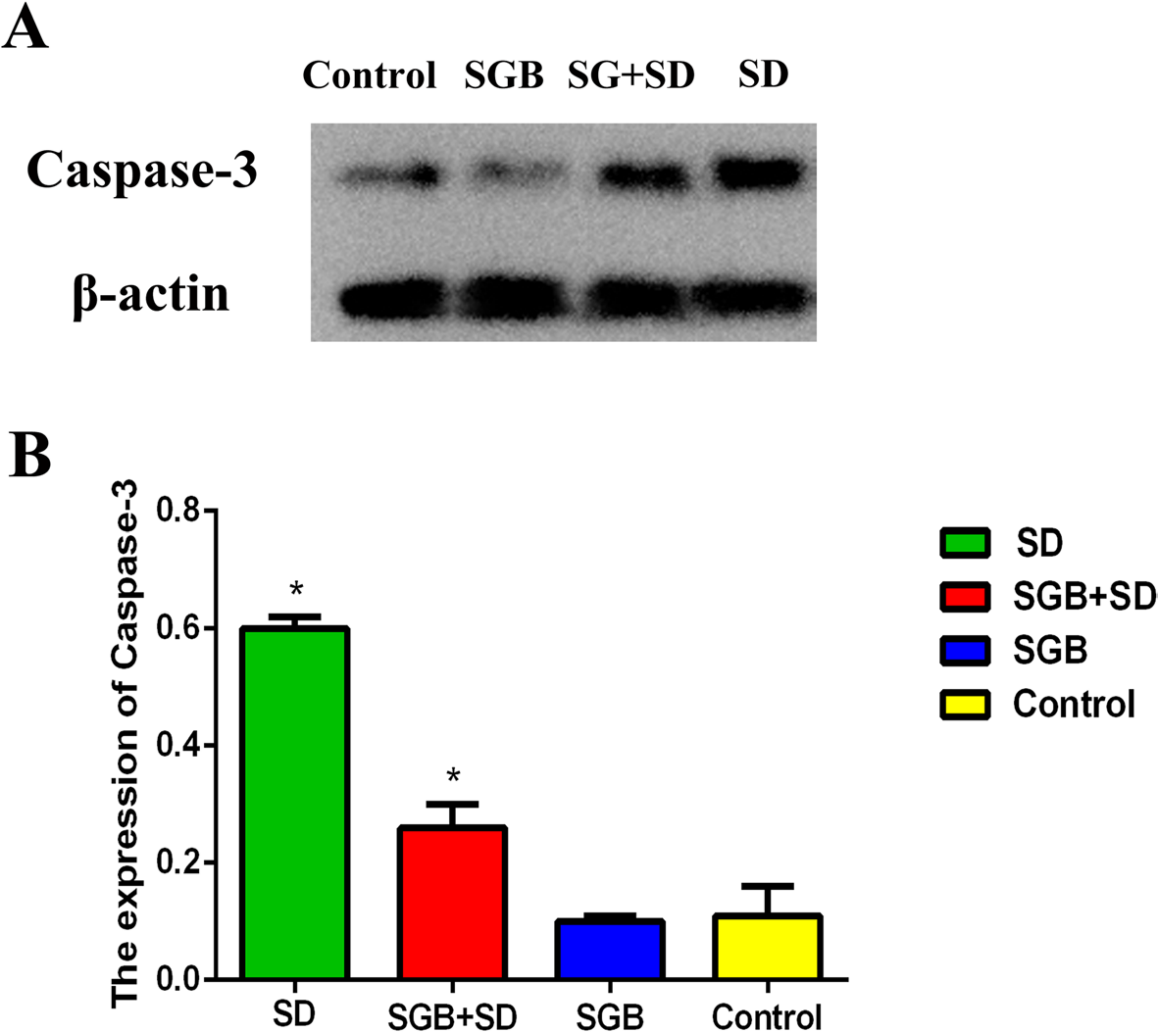
Results of western blotting detection of Caspase-3 expression of each group of rats (**A**) and internal reference expression in hippocampal tissue (**B**) (n = 8^−^x,±s). Note: *P < 0.05, as compared with the Control group

The western blot analysis showed that the relative expression of Caspase-3 protein in hippocampus of SD group was significantly higher (*P* < 0.05) than that of control group (Fig. 4A). The relative expression of Caspase-3 protein in hippocampus of SD + SGB group was significantly lower (*P* < 0.05) than that of SD group (Fig. 4B). There was no significant difference in the relative expression of Caspase-3 protein between SGB group and control group (*P* = 0.667 > 0.05) (Fig. 4B).

### Right stellate ganglion block after sleep deprivation alleviated the cellular injury and neuronal damage in hippocampus of rats

**Fig. 5:**
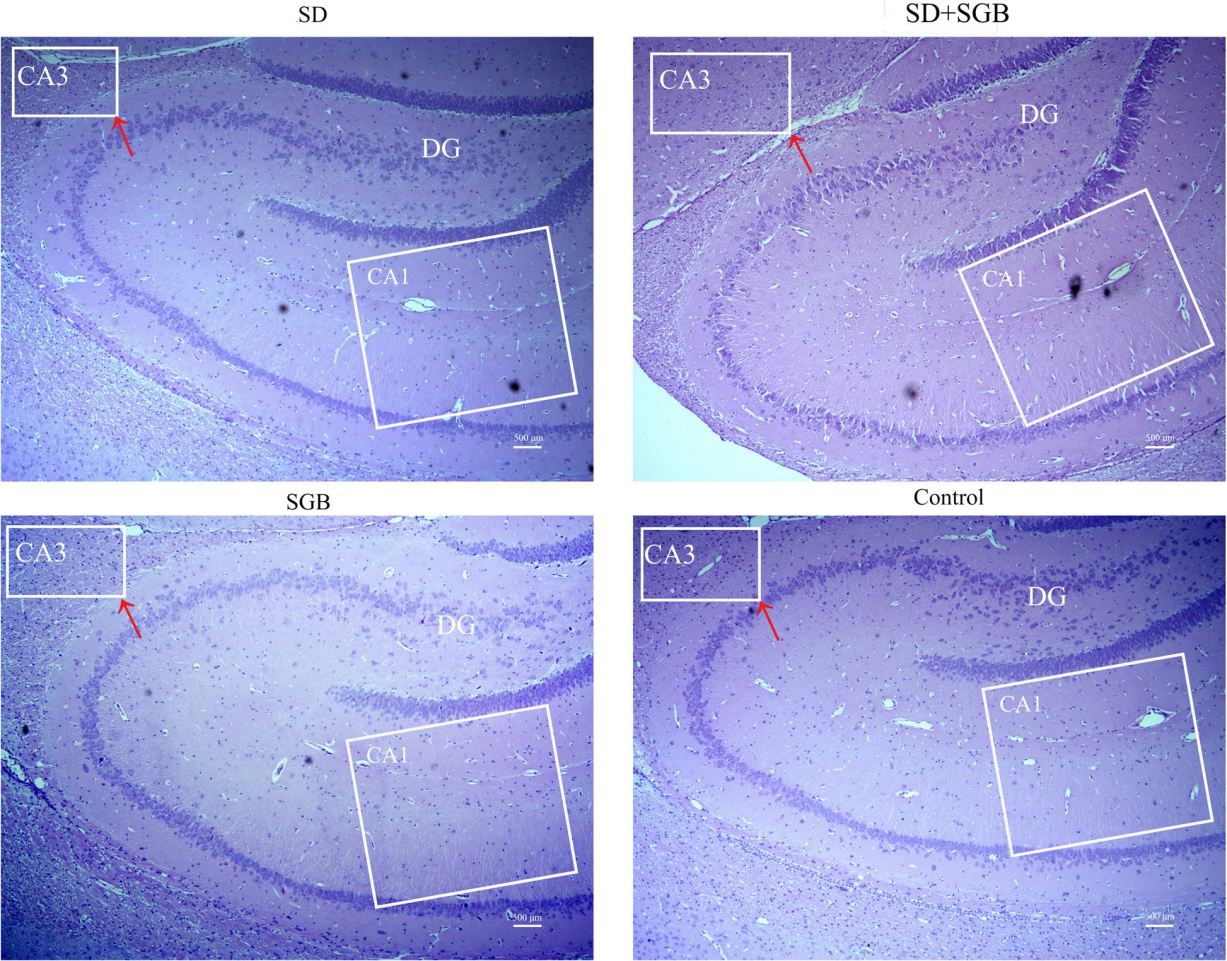
Results of HE stains in hippocampal vertebral cells of rats in each group (200×). Note: The pathological sections of rat hippocampus are shown. The degree of stress damage of hippocampal vertebral neurons in SD group was significantly higher than that in C group; SD + SGB group was significantly lower than that in SD group. The degree of stress damage of hippocampal vertebral nerve cells in SGB group was less than that in C group

The histochemical analysis of hippocampal tissue showed that the degree of stress damage and injury of hippocampal neurons in SD group was serious, especially the vertebral neurons in hippocampal CA3 region (Fig. 5). The arrangement of hippocampal vertebral cells was disordered, the cells became smaller, the morphology was irregular, the stroma was loose, the nucleus showed pyknosis and the structure of some cells was not clear. Compared with the SD group, the neuronal damage in the CA3 region of the hippocampus of the SD + SGB group was significantly improved, the cell arrangement was more orderly, the distribution was more uniform, and the structure was clear. The results of HE stains of hippocampal neurons in SGB group and group were similar, and the neurons of vertebral body were arranged in layers and distributed evenly.

## Discussion

Sleep is an important physiological process to maintain the normal activity of the human body, and it has almost the same physiological significance as the breathing and heartbeat of the body. In recent years, there are many excellent reviews in the literature dealing with the basic concepts of lack of sleep that can lead to learning and memory impairment [3, 4, 7, 33, 34]. Specifically, sleep deprivation may cause cognitive impairment by injury of hippocampal tissue [35, 36], excessive inflammatory reaction of neurons during passive arousal [37], disorder of pineal gland secretion, sudden decrease of MT [38], apoptosis of nerve cells [39], etc. Therefore, the pathological changes, inflammatory factor expression, apoptosis and serum MT content in hippocampal tissue were detected in this experiment. Compared with control group, SD group rats escape latency was significantly prolonged, and the number of crossing platforms and the percentage of quadrant time of the original platform were significantly reduced, indicating that the sleep deprivation model established in this experiment was successful and effective. Studies have found that bilateral SGB can cause bilateral recurrent laryngeal nerve paralysis and airway obstruction [40]. Left stellate ganglion block can damage left ventricular function and reduce cardiac stroke volume [41]. In acute coronary occlusion model, it is found that left stellate ganglion block does not improve the balance of oxygen supply and demand, and may increase the risk of myocardial ischemia, while right stellate ganglion block can regulate the balance of oxygen supply and demand [42]. Right stellate ganglion block has the effect of stabilizing ECG activity [43]. In the model of spontaneous hypertension, right stellate ganglion block can reduce cardiomyocyte apoptosis and reverse left ventricular remodeling by affecting apoptosis related gene regulatory proteins [44, 45]. Therefore, right stellate ganglion block was used in this experiment.

The escape latency of rats in SD + SGB group was significantly shorter than that in SD group, while the number of crossing the platform and the percentage of residence time in the quadrant of the original platform were higher than those in SD group, indicated that the multiple right-side stellate ganglion block can effectively relieve the function of learning and memory caused by the sleep deprivation. Our results are consistent with previous studies which demonstrated that the SGB can improve the post-operative cognitive dysfunction by excessive expression of AMPK, which is caused by the down-regulation of the stress-induced trauma of the operation and the inhibition of excessive activation of astrocytes, and regulating cerebral oxygen metabolism [46, 47].

After 96 h sleep deprivation, the weight of SD rats in the experimental group lost weight, and the weight of the control group increased normally. This finding was contrary to another previous study report which mentioned that the sleep deprivation rats gain normal weight [48]. The discrepancy in body weight analysis may be due to the fact that when the rats are in the process of sleep deprivation, the splashed water soaked the feed when it fell off the platform, causing the taste of the feed to deteriorate, and the lack of food intake in the rats led to weight loss. Another reason could be because of continuous complete sleep deprivation which might have affected the gastrointestinal function of rats, resulting in weight loss. Notably, the difference in body weight was insufficient to make a significant contribution to the improvement in cognitive performance. The mechanism of improved cognition by ganglion blockade is to a greater extent the alleviation of inflammatory stimuli from the intracranial. Of course, in order to clarify these reasons, we need to design and carry out further experiments and follow-up research.

It was found that the secretion of MT from pineal gland decreased gradually with the prolongation of sleep deprivation time. During sleep deprivation, rats were forced to awaken, and the pineal function of synthesizing and secreting MT was inhibited, so the content of MT in pineal gland decreased sharply [49], which is consistent with the results of this experiment. The content of serum MT in SD + SGB group was significantly higher than that in SD group, which indicated that multiple right stellate ganglion block could effectively alleviate the decrease of MT secretion induced by sleep deprivation.

In this experiment, the content of IL-6, IL-1β in hippocampus of SD + SGB group was significantly lower than that of SD group after sleep deprivation, which indicated that right stellate ganglion block could effectively alleviate the hyperinflammation in rats with sleep deprivation. Identical conclusions were obtained in studies where the effect of unilateral cervical sympathetic nerve block on early inflammatory response in patients with severe trauma. It was found that right stellate ganglion block could inhibit the early inflammatory reaction of severe trauma by regulating pro-inflammatory cytokines IL-1 β, IL-6 and TNF-α [50]. Moreover, Yang X found that stellate ganglion block may be involved in the regulation of neuroendocrine and immune system dysfunction in traumatic brain injury, the mechanism is to regulate NF- κ B protein, IL-1 β and TNF- α in lymphocytes through neuroendocrine immune system, CGRP, and inhibit the early excessive inflammatory reaction of traumatic brain injury, thus protecting the brain function of traumatic brain injury [51].

The results of Western blotting showed that the expression of Caspase-3 protein in hippocampus of SD + SGB group was significantly lower than that of SD group, which indicated that administration of multiple right stellate ganglion block could effectively alleviate the apoptosis of hippocampal neurons induced by sleep deprivation. This is consistent with the observation from Chen Y’s study of the spontaneous hypertensive rat model, right stellate ganglion block could significantly decrease the expression of Bax and increase the expression of Bcl-2/Bax in spontaneously hypertensive rats, which confirmed that right stellate ganglion block could inhibit cardiomyocyte apoptosis by regulating apoptosis-related gene regulatory proteins [52]. The results of HE stains showed that the degree of stress damage of hippocampal neurons in SD + SGB group was significantly lower than that in SD group, indicating that right stellate ganglion block could alleviate the hippocampal injury induced by SD.

The main experimental advantages of this study were indicated by the experimental results of the rat water maze that are affected by the memory function of the rats on the one hand and the physical strength of the rats on the other hand. Therefore, the SGB group was established in this experiment, and the water maze exploration experiment was also performed in the SGB group. The memory function of the SGB group was similar to that of the Control group. In other word, the decrease of spatial memory function in SD group was caused by memory impairment rather than lack of physical strength. In this experiment, the SGB group and the Control group adopt the large platform water environment method, which is close to the environmental factors of the multi-platform water environment and can eliminate the differences between the groups caused by environmental factors. Furthermore, the rats were allowed to acclimate in the water environment for 1 week before the experiment, reducing the additional stress factor caused by placing the rats in the sleep deprivation box. This experiment used a behavioral platform and found that prophylactic administration of right stellate ganglion block in sleep deprivation rats can effectively alleviate the spatial learning and memory dysfunction induced by sleep deprivation in rats and its possible related mechanisms, providing a preventive treatment for those who have to work overtime and stay asleep and cause memory loss. It is worthwhile mentioning that ultrasound-guided stellate ganglion block is simple and non-invasive. In addition, it has been found that ultrasound-guided stellate ganglion block can block stellate ganglion directly and accurately, greatly reducing the stellate ganglion under blind test. The side effects of blockade increase the safety, accuracy, reliability and effect of stellate ganglion block, which is worth promoting.

There are some limitations of this study. This experiment used an sleep deprivation model for the experiment, and the rats were sacrificed immediately after the experiment, and therefore unable to observe the long-term effects of stellate ganglion block on sleep deprived rats. In this study, a sleep deprivation model was performed, and further studies on stellate ganglion block in rats with chronic sleep deprivation can be performed later. This experiment is only a simple observation of the possible mechanism of stellate ganglion block in sleep deprived rats rather than the exact mechanism, which is also the future direction we need to explore later.

## Conclusions

In summary, the right stellate ganglion block can effectively alleviate spatial learning and memory dysfunction induced by SD in rats. The mechanism may be related to alleviate the excessive inflammatory reaction and neuronal apoptosis in hippocampus and alleviate the decrease of MT.

## Supplementary Information

**Additional file 1.**

## Data Availability

The data used to support the findings of this study are available from the corresponding author upon request.
