# Supplementary figures and images for: Right stellate ganglion block improves learning and memory dysfunction and hippocampal injury in rats with sleep deprivation
Source: BMC Anesthesiol. 2021 Nov 8;21:272. doi: 10.1186/s12871-021-01486-4 (PMC8574040; doi:10.1186/s12871-021-01486-4)

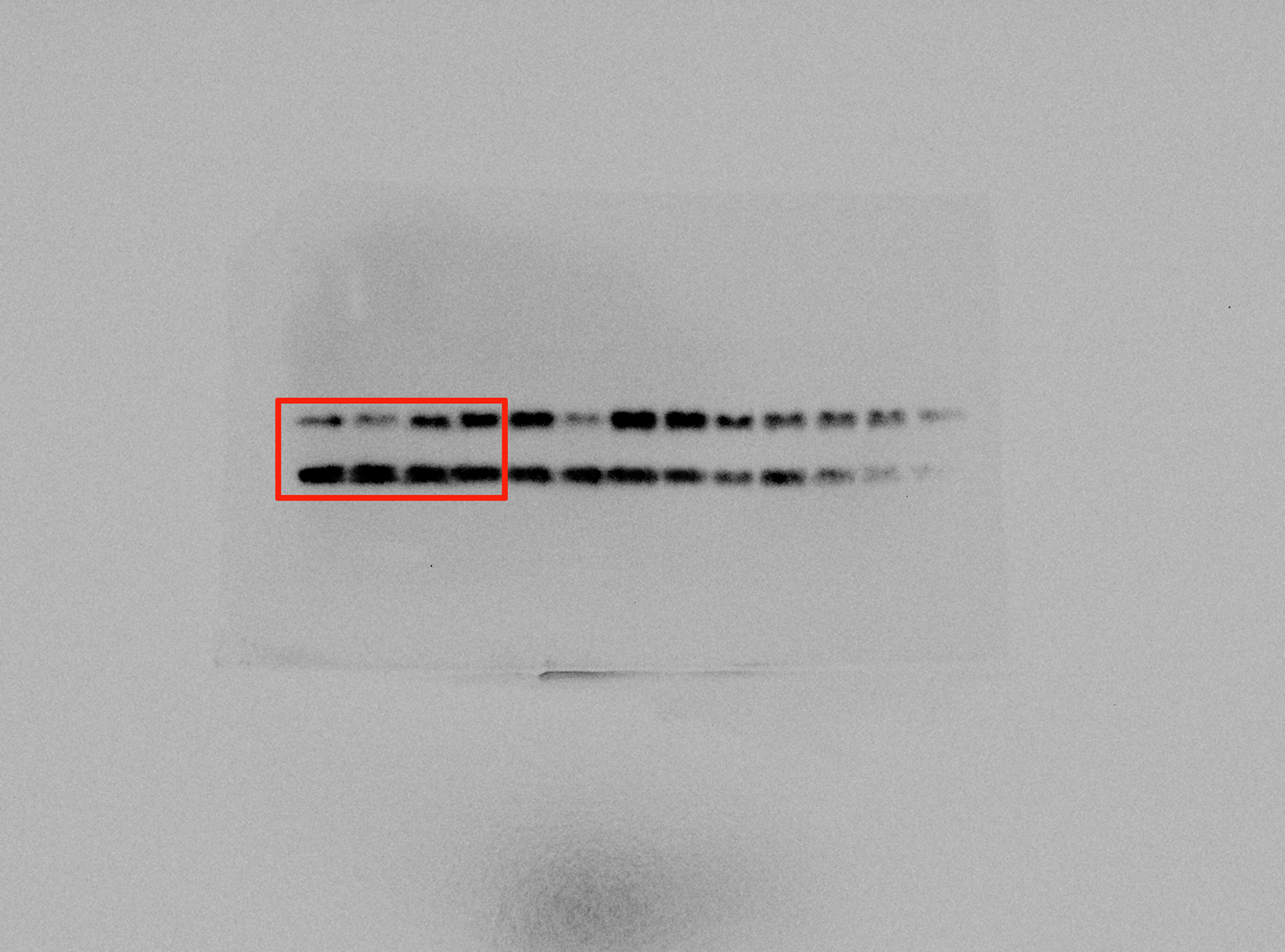

Supplement: Supplementary file 1 — Additional file 1. [file 12871_2021_1486_MOESM1_ESM.tif]
